# Integrating Psychological Services Into Community-Based Clinics

**DOI:** 10.1093/geroni/igab046.2019

**Published:** 2021-12-17

**Authors:** Lindsey Jacobs

**Affiliations:** The University of Alabama, The University of Alabama, Alabama, United States

## Abstract

In Alabama, where mental health stigma is a critical barrier to care, integrated behavioral health services are vital to address the mental health needs that underlie substance use disorder (SUD) and opioid use disorder (OUD). Since October 2019, our team has developed partnerships with one rural and two peri-urban primary care clinics to offer behavioral health services with an emphasis on SUD/OUD prevention, screening, and treatment. The patient populations receiving services at these three facilities are under-resourced with multiple disadvantages placing them at risk for morbidity, mortality, SUD/OUD, and poor behavioral and mental health outcomes. Behavioral health services have been delivered primarily via telehealth due to the COVID-19 pandemic. This presentation will describe the process, current status, and future goals for implementing integrated behavioral health care, with a focus on identifying the barriers and facilitators during the COVID-19 pandemic era.

